# Topoisomerase IV tracks behind the replication fork and the SeqA complex during DNA replication in *Escherichia coli*

**DOI:** 10.1038/s41598-020-80043-4

**Published:** 2021-01-12

**Authors:** Emily Helgesen, Frank Sætre, Kirsten Skarstad

**Affiliations:** 1 Department of Microbiology, Molecular Microbiology, Oslo University Hospital, P.O. Box 4950, 0424 Oslo, Norway; 2 Department of Clinical and Molecular Medicine, Faculty of Medicine and Health Sciences, Norwegian University of Science and Technology, Trondheim, Norway; 3 Present Address: Department of Pathology, Institute of Clinical Medicine, University of Oslo, Oslo, Norway

**Keywords:** Replisome, Chromosome segregation

## Abstract

Topoisomerase IV (TopoIV) is a vital bacterial enzyme which disentangles newly replicated DNA and enables segregation of daughter chromosomes. In bacteria, DNA replication and segregation are concurrent processes. This means that TopoIV must continually remove inter-DNA linkages during replication. There exists a short time lag of about 10–20 min between replication and segregation in which the daughter chromosomes are intertwined. Exactly where TopoIV binds during the cell cycle has been the subject of much debate. We show here that TopoIV localizes to the origin proximal side of the fork trailing protein SeqA and follows the movement pattern of the replication machinery in the cell.

## Introduction

Proper segregation of newly replicated DNA is essential for the viability and genetic stability of all cell types. Due to the superhelical nature of DNA molecules, topology challenges are inevitable during the process of DNA replication, as the template strands are separated and duplicated. More specifically, tension arises in front of the replication machinery (hereafter called the replication fork) as the parental DNA strands are pulled apart, which results in the formation of positive supercoils (overwinding)^1,2^. Some of these positive supercoils may diffuse towards the newly replicated DNA molecules behind the replication fork, and the replication fork most likely rotates to alleviate some of the topology tension piling up ahead^3^. As a consequence, the newly replicated DNA molecules become intertwined, and this type of entanglement is typically referred to as precatenanes^1–4^. Without the removal of precatenane linkages it becomes impossible for the cell to segregate the DNA prior to cell division. Highly specific mechanisms therefore exist to resolve the topological issues that arise during DNA replication, and at the core of these mechanisms we find the enzymes categorized as type II topoisomerases^2^.

In *Escherichia coli* two type II topoisomerases are involved in enabling both DNA replication and timely DNA segregation, namely Gyrase and Topoisomerase IV (TopoIV). Both of these enzymes work by first performing a transient double strand break in one molecule, then leading a second DNA duplex through the cut and lastly, resealing the cut. They are heterotetrameric structures consisting of GyrA and GyrB subunits or ParC and ParE subunits for Gyrase and TopoIV, respectively. The GyrA/ParC subunit contains the DNA binding and catalytical properties of the enzyme, whereas ATP binding resides in GyrB/ParE^5^. It is now generally well recognized that Gyrase acts in front of the replication fork to remove excess positive supercoiling to support fork progression, whereas TopoIV mainly removes precatenane linkages after replication to facilitate DNA segregation^6–9^. However, there has been much debate concerning the precise timing and localization of TopoIV action. It has been suggested that TopoIV activity is limited to the D-period (when a round of DNA replication is completed) and that TopoIV localizes mainly at the terminus^10^. It has also been indicated that the catalytically active TopoIV molecules bind in clusters at the origins, where they are recruited and stimulated by MukB, an SMC (structural maintenance of chromosomes) protein^11–13^. Moreover, there is a time lag of 10–20 min (depending on media and growth rate) between replication of the DNA and segregation of the newly replicated DNA, which is termed the “cohesion period”^6,8,9,14^. Whether this means that TopoIV does not immediately gain access to the DNA after replication (i.e. that precatenanes hold the homologous DNA together), or if other factors such as proteins bridging the DNA is causing this delay, is not completely understood.

In this work we have sought to elucidate the localization and movement of TopoIV with respect to the replication fork and a fork-trailing protein named SeqA. SeqA is a negative modulator of replication initiation, which binds to newly replicated, hemimethylated GATC-sites^15–17^. SeqA forms multimeric structures which trail the replication forks dynamically, always binding to the newest DNA^18–21^. The SeqA-DNA complexes are large and typically encompass 100 kb of DNA. We have previously found that the SeqA multimer binds at a distance from the replisome (on average 200–300 nm)^22^. The newly replicated DNA molecules were found to be kept close together on this stretch, i.e. they were cohesed. The localization of the cohesed DNA and the replisomes in the cell were visualized by utilizing fluorescently tagged SeqA (SeqA-YFP) and replisome proteins (SSB-CFP), respectively.

We find here that fluorescently tagged TopoIV (ParC-mKate2) exhibits a localization pattern throughout the cell cycle compatible with the model that TopoIV trails SeqA and the replisome during replication. Moreover, the average distance between TopoIV and the replisome is always larger than that between SeqA and the replisome. This indicates that TopoIV is indeed excluded from binding to the DNA immediately after its replication. Inhibition of TopoIV using a fluoroquinolone antibiotic, Ciprofloxacin, lead to an increased distance between SeqA and TopoIV, presumably because the TopoIV molecules become “stuck” in DNA ternary complexes, thereby lagging even further behind the replication machinery.

## Results

### TopoIV most likely trails SeqA during DNA replication

In order to investigate the localization of TopoIV with respect to the replication fork and the newly replicated DNA, we constructed a strain which contains fluorescent tags on the single-stranded binding protein (SSB-CFP) present at the replisome, on SeqA (SeqA-YFP) and on TopoIV (ParC-mKate2). The cells exhibited a normal growth rate and cell cycle compared to the wild type background, i.e. they were able to successfully complete DNA replication and had no observable segregation issues (see Table 1 for generation times and cell cycle parameters and Fig. S1 for flow cytometry histograms).

**Table 1:** Cell cycle parameters for strains grown in acetate medium at 28 °C (averages from at least three separate experiments ± SEM).

Strain	Doubling time	C-period	D-period	Initiation
AB1157 Parent	194 ± 26	103 ± 11	122 ± 6	164 ± 47
EH29 SeqA-YFP SSB-CFP ParC-mKate2	184 ± 12	104 ± 10	126 ± 7	139 ± 25
EH32 gyrA^L83Y87^	177 ± 42	119 ± 54	73 ± 44	93 ± 92
EH34 SeqA-YFP ParC-mKate2 gyrA^L83Y87^	156 ± 36	96 ± 23	86 ± 11	68 ± 58

**Figure 1:**
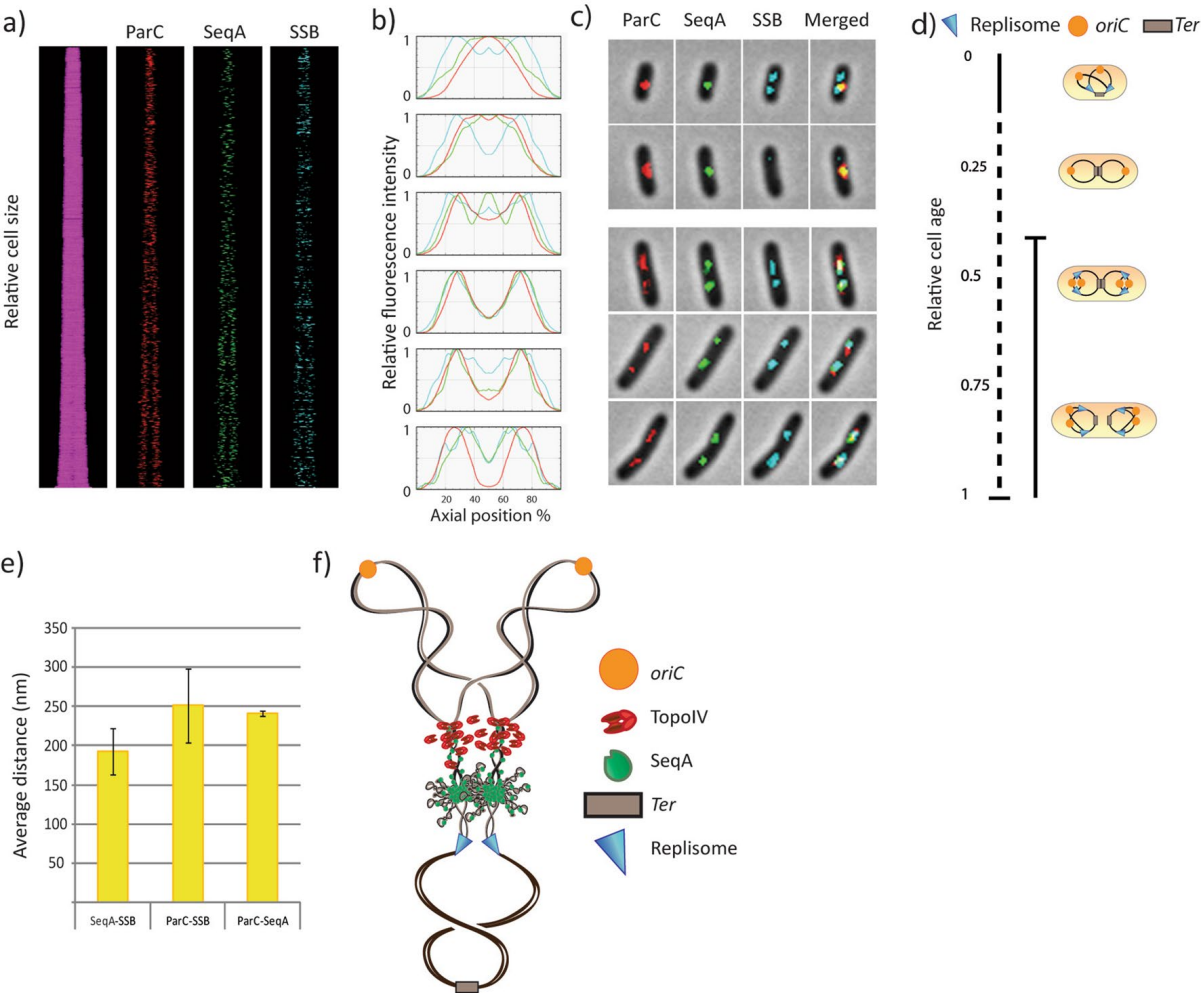
Fluorescence microscopy indicates that TopoIV trails the replication forks and primarily decatenates the precatenanes farthest from the replisome. (**a**) Kymographs showing fluorescent foci of TopoIV (ParC-mKate2), SeqA (SeqA-YFP) and replisome (SSB-CFP) in cells stacked horizontally according to cell size (smallest cells top and largest cells bottom). (**b**) Plots of relative fluorescence intensity (Y-axis) according to position on the cell long axis (X-axis) in groups of cell sizes/ages from smallest (top) to largest (bottom). (**c**) Images of representative cells from snap-shot microscopy arranged according to approximate cell cycle stage. (**d**) Schematic cartoon showing cell cycle progression and DNA content according to cell size. The solid black line represents the replication period (C-period) with initiation around a relative cell age of 0.42, whereas the stippled black line represents the post replication period (D-period). Cell cartoons are shown to indicate DNA content and cell cycle progression. (**e**) Plot showing the average distances (nm) between SeqA-SSB, ParC-SSB and ParC-SeqA in the cells from three independent experiments. Error bars are included in the plot. > 600 cells were analyzed. (**f**) Hypothetical cartoon showing the relative localization of the replisomes (cyan triangles), SeqA molecules (green) and TopoIV molecules (red) on an actively replicating chromosome, about halfway through the replication cycle. The old DNA is shown as black lines, the newly synthesized DNA as grey lines, the origins (*oriC*s) in orange and the terminus region in brown.

We grew the cells in poor medium (acetate medium) to early exponential phase (OD ~ 0.15) and investigated the living cells with snap-shot fluorescence microscopy. The poor medium was chosen in order to generate a simple cell cycle in which each chromosome harbors no more than two replication forks. This enables a more straightforward interpretation of images, compared to a situation in which the cells perform multi-fork replication (more than two forks per chromosome). The images were subjected to analysis with Coli Inspector (see “Methods” for details) in order to assess the positioning of fluorescent foci. From kymographs of the fluorescent foci (in which the cells are stacked according to cell size) (Fig. 1a) and plots of relative fluorescence intensity according to position along the cell long-axis (Fig. 1b), we found that TopoIV had a localization pattern that resembled that of SeqA and the replisome (see Fig. 1c for representative images and Fig. 1d for cell cycle analysis). The pattern was verified by live cell time-lapse imaging, in which the cells were imaged over a time course of 60 min (Fig. S1). These data support the model that TopoIV trails the replication machinery to ensure processive removal of precatenanes, and that it is not restricted to performing decatenation after replication termination.

As observed in previous studies the replisome appears to be more dynamic compared to the replisome-trailing SeqA structures, as one replisome focus more frequently represents one replication fork at each of the quarter positions in cells growing with one replicating chromosome^22,23^ (see young cells in Fig. 1a–c) compared to SeqA which stays at mid cell (thus representing four strands of newly replicated DNA). We found that TopoIV had a localization pattern which was more similar to that of SeqA than to that of the replisome, which is especially prominent in the newborn cells harboring SeqA and TopoIV at midcell (Fig. 1b,c, top panels). This may indicate that TopoIV is closer to SeqA than to the replisome. To further elucidate this scenario we measured the distances between the three fluorescently tagged structures using high-throughput image analysis scripts described previously^22,24^. Briefly, after processing of the images, the script measures the distances between the highest intensity pixels from each channel/focus, in which the highest concentration of molecules are likely to be situated. From three separate experiments we found that the average distance between SeqA and the replisome was always less than that between TopoIV and the replisome (see average values Fig. 1e). This finding suggests that TopoIV binds on the origin-proximal side of SeqA and the replisome (Fig. 1f).

### The distance between SeqA and TopoIV increases when TopoIV is inhibited by ciprofloxacin

The group of antibiotics termed fluoroquinolones is known to bind and inhibit Gyrase and TopoIV by forming a ternary complex with these enzymes and DNA. Upon drug interaction Gyrase/TopoIV remains as a “frozen” adduct on DNA after the cleavage step, and is unable to reseal the double-strand ends after strand passage^25^. We decided to use the fluoroquinolone Ciprofloxacin to shed more light on the positioning of TopoIV during replication. If TopoIV is localized between the SeqA complex and the replisome, one would expect to observe a perturbation of SeqA focus formation upon inhibition of TopoIV, since SeqA may “collide” into the frozen adducts that occupy the space necessary for SeqA binding and multimerization. If, on the other hand, TopoIV trails SeqA, it would be expected that the distance between the SeqA complex and TopoIV increases compared to the untreated control, as the TopoIV-Ciprofloxacin adducts will be lagging behind on the origin-proximal side of the SeqA complex.

To ensure that only TopoIV would be targeted in our experiments, we used a strain which contains two mutations in the GyrA subunit of Gyrase (L83 and Y87)^26^, rendering Gyrase insensitive to fluoroquinolones, in addition to the fluorescently tagged SeqA (SeqA-YFP) and TopoIV (ParC-mKate2) constructs. The cells were grown in acetate medium to early exponential phase (OD ~ 0.15) and either imaged directly (as described in the previous section) or treated with 0.1 µg/ml Ciprofloxacin for 45 min prior to imaging. The low concentration of Ciprofloxacin was used in order to avoid double-strand breaks, SOS-response and cell cycle arrest, as this would generate a scenario in which the replication forks are not proceeding as normal. The incubation time was set to less than the time it takes for one round of replication to avoid that newly fired replication forks collide into TopoIV adducts formed in the previous round of replication.

**Figure 2:**
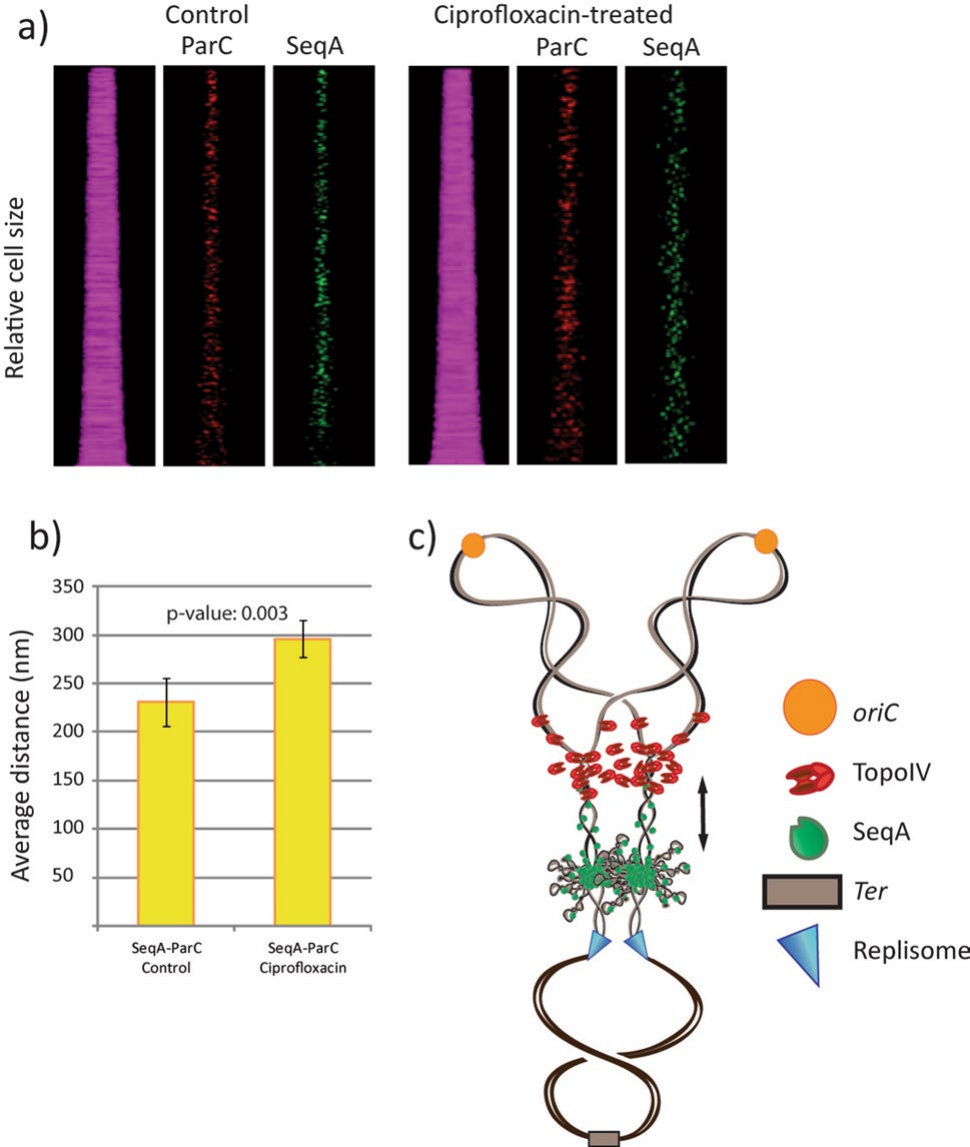
Treatment with the antibiotic Ciprofloxacin supports the idea that TopoIV activity trails the replication fork on the origin-proximal side of the “cohesion window”. (**a**) Kymographs showing fluorescent foci of TopoIV (ParC-mKate2) and SeqA (SeqA-YFP) in cells stacked horizontally according to cell size (smallest cells top and largest cells bottom). Untreated cells are shown to the left whereas cells treated with Ciprofloxacin (0.1 µg/ml) for 45 min are shown to the right. The strain EH32 was used, which contains mutations in Gyrase (L83Y87) rendering Gyrase insensitive to Ciprofloxacin. (**b**) Plot showing the average distances (nm) between SeqA and TopoIV (ParC) in untreated (left) and Ciprofloxacin treated (right) cells from three independent experiments. Error bars are included in the plot. > 700 cells were analyzed. The p-value for increase in SeqA-ParC distances in Ciprofloxacin treated cells is indicated in the plot and was calculated using a paired, one-tailed T-test on average distances from three independent experiments. (**c**) Hypothetical cartoon showing how TopoIV (red) may lag farther behind SeqA (green) when inhibited by Ciprofloxacin on the newly replicated DNA. The old DNA is shown as black lines, the newly synthesized DNA as grey lines, the origins (*oriC*s) in orange and the terminus region in brown.

Image analysis showed that the localization pattern of SeqA and TopoIV was different in the Ciprofloxacin-treated cells compared to the untreated control (Fig. 2a). This is not surprising, considering that the ability of TopoIV to properly facilitate decatenation and segregation is compromised. However, the Ciprofloxacin-treated cells had no problem with SeqA focus formation, and when measuring the SeqA-TopoIV distances in the cells we found that the average distance was indeed increased in the Ciprofloxacin-treated culture (r = 0.70, p = 0.033) (Fig. 2b). A schematic model is depicted in Fig. 2c, showing how the Ciprofloxacin-bound TopoIV complexes may become stuck in the DNA and lag behind SeqA, thus leading to an increased SeqA-TopoIV distance.

## Discussion

**Figure 3:**
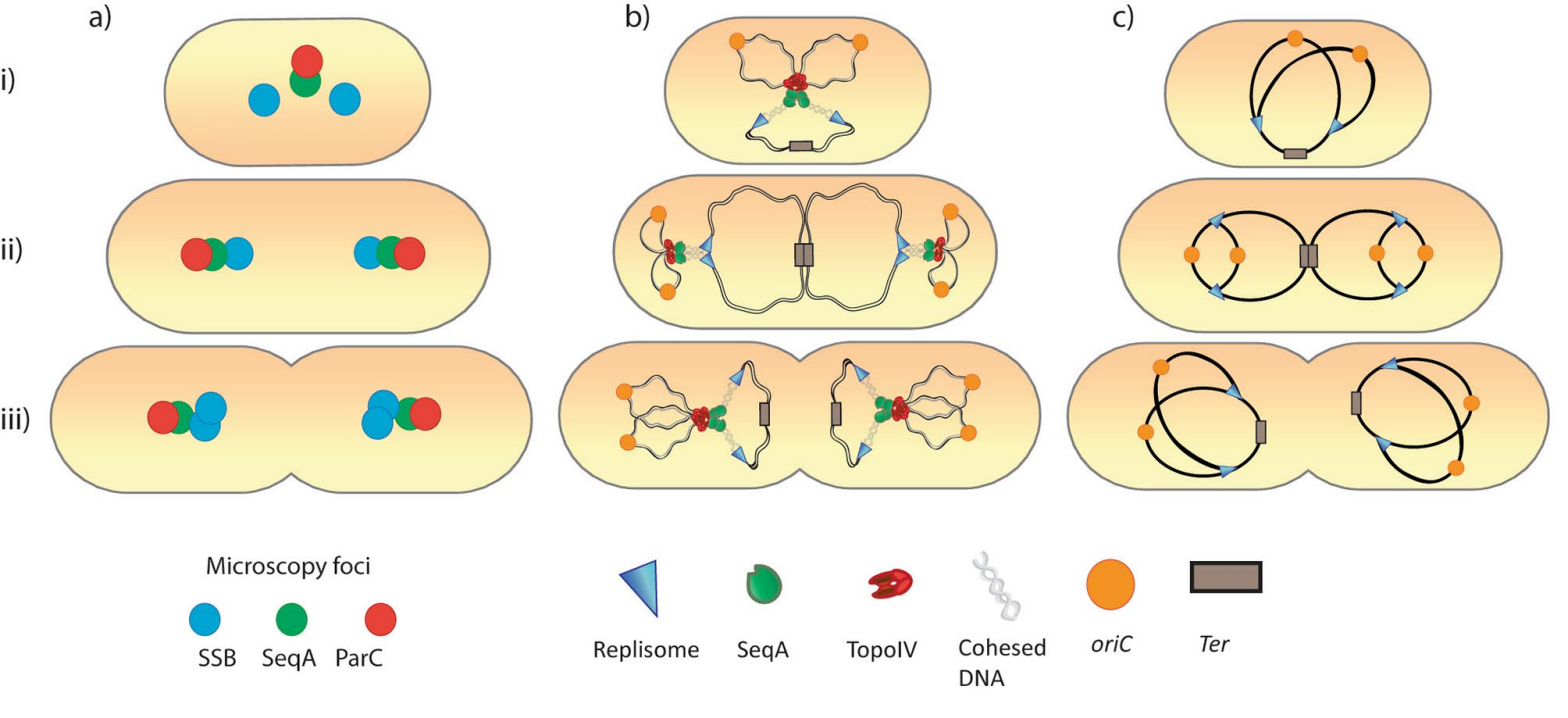
Schematic model figures showing approximate positioning of SeqA, TopoIV and the replisome on the DNA at different stages in the cell cycle according to microscopy foci and cell cycle analysis. (**a**) Cartoons showing the positioning of fluorescent foci of TopoIV (ParC), SeqA and the replisome (SSB) in the cell during the cell cycle, as seen by fluorescence microscopy (general trend). (**b**) Cartoons showing the positioning of DNA and organization of replication forks during the cell cycle. The placement of TopoIV, SeqA and the replisome is indicated. Hypothetical positioning of *oriC* and terminus is also indicated. The old (unreplicated) DNA is shown as black lines, whereas the newly replicated DNA is shown as grey lines. The “cohesion window” between SeqA and the replisome is represented by transparent, intertwined lines of DNA. (**c**) Simplified version of (**b**) more accurately representing cell cycle stage, replication fork progression and DNA content. Organization of DNA at the replication fork and positioning of SeqA and TopoIV is not indicated. Upper panels (i): newborn cells. Middle panels (ii): middle-aged cells in which initiation of replication has recently occurred. Lower panels (iii): old cells that are about to divide.

The localization and action of Topoisomerase IV on the bacterial chromosome has been a subject of much debate. It is known that DNA segregation occurs gradually during the DNA replication phase (with a short time lag with respect to replication)^14^ and it is known that TopoIV has an important role in unlinking the newly replicated DNA molecules to enable segregation^6,9^. It appears sensible that TopoIV molecules trail the replication machinery to make this possible. However, previous experimentation has suggested quite different scenarios. One suggestion involved localization of TopoIV at the terminus and activity carried out only in the D-period (after all replication is completed)^10^. Other studies indicated localization of TopoIV in clusters at *oriC*, where a key interaction with MukB was required for both localization and activity^11–13^. In this study we sought to clarify this confusing subject by microscopy analysis of a strain with fluorescently tagged replisome (SSB-CFP), SeqA (SeqA-YFP) and TopoIV (ParC-mKate2). We find that the localization pattern of TopoIV during the cell cycle is strikingly similar to that of the replication machinery and the fork trailing protein SeqA (Fig. 1a–c). This strongly suggests that TopoIV follows the movement of the replication fork during the cell cycle. We also find that TopoIV binds further away from the replisome than the SeqA complex does (Fig. 1e) and that the distance between TopoIV and SeqA increases after inhibitory treatment of TopoIV with Ciprofloxacin (Fig. 2). These data indicate that TopoIV binds on the origin-proximal side of the SeqA complex. Figure 3 summarizes our findings by showing the general localization trend of fluorescent foci (as seen by microscopy) in the cell (Fig. 3a) and the extrapolation of this data (combined with analysis of cell cycle progression and DNA content) to illustrate the localization of the replisome, SeqA and TopoIV on the replicating chromosome with respect to each other and to *oriC* and terminus (Fig. 3b). A simplified cartoon of the DNA and replication fork content at different stages of the cell cycle is included in Fig. 3c.

By flow cytometry analysis of DNA content (as described in^27^) we found that the newborn cells contain one replicating chromosome in which the two replication forks are more than halfway through the chromosome and are approaching the terminus (Fig. 3 upper panels (i)). In these cells SeqA and TopoIV are localized relatively close together at mid-cell (Fig. 1a–c and illustrated in Fig. 3). From previous studies we know that cells which are about to terminate replication of a chromosome have already segregated their two origins to the respective quarter positions in the cell^22,24^ (illustrated in Fig. 3b top panel (i)). Since TopoIV is not found at quarter positions in these cells, we suggest that TopoIV is not exclusively found in clusters associated with MukB at the origins, as inferred in^11,13^. In this study we only find evidence of TopoIV close to origins at or recently after initiation of replication (when also SeqA and the replisome are close to origins, Fig. 3b ii). Recently, it was found that the MukB-TopoIV interaction at origins in fact promoted DNA condensation and did not involve any catalytic activity of TopoIV^28^. It may therefore be that TopoIV bound to MukB at origins does not contribute to resolution of precatenanes, but instead has other roles.

As previously mentioned, a time lag of about 10–20 min has been observed between replication of the DNA and segregation of the DNA in *E. coli*^6,8,9,14^. This period is denoted the “cohesion” period, because the two newly replicated molecules of DNA behind each fork are localized very close together (see “cohesed DNA” in Fig. 3b). From our previous studies we have found that this distance is less than 30 nm^22^, and others have found that the two DNA double strands are close enough to support homologous recombination^8^. From analyses in this study it appears likely that TopoIV does not gain access to the DNA directly after replication, since it is positioned at a distance from the replisome and behind SeqA. It seems therefore likely that the new DNA molecules are bound together by precatenanes in this time period and that delayed action of TopoIV is causing the much discussed cohesion period. This is also supported by other studies^8,9^.

How the cohesion window is maintained is currently not understood. One possible explanation could be that this stretch of DNA is occupied by other proteins, which inhibit TopoIV binding. For instance, high concentrations of SeqA have been shown to inhibit TopoIV activity in vitro^29^, and this finding correlates with in vivo studies showing increased cohesion period upon SeqA overexpression^6^. However, it could also be that the topology of the precatenated DNA directly behind the replication fork is not optimal for TopoIV binding and that TopoIV require some initial topology modifications for proper binding and action. Follow up studies will hopefully elucidate unanswered questions concerning maintenance and control of the cohesion period.

## Methods

### Strain construction

All strains used in experiments are derivatives of the *E. coli* K-12 strain AB1157^30^ and are listed in supplementary Table S1. Localization studies of SeqA were done with cells containing the yellow fluorescent protein (YFP) fused to the C-terminal end of SeqA^31^. The *seqA-yfp* gene was expressed from the endogenous chromosomal promoter. The YFP protein was from^32^ and connected to SeqA via a four-amino acid linker^18^. Studies of SSB localization were with cells containing the SSB-CFP allele inserted in place of the *E. coli lamB* gene and was kindly provided by A. Wright (G. Leung et al., unpublished)^22^. The cells contained the wild-type *ssb* gene on the chromosome. The SSB-based marker has been verified to represent the localization of the replisome, as it perfectly colocalizes with another replisome unit, namely HolC^22^. The fluorescent version of ParC was constructed in this study. Briefly, the *mKate2* gene was PCR amplified from the plasmid pTEC20^33^, subcloned via pGEM-T easy (Promega) and inserted upstream of a chloramphenicol resistance cassette in the plasmid pSF36 (pUC19 + cm-FRT-*HindIII*) yielding plasmid pEH04. Primers with 50 bp homology to the C-terminus of *parC* and 50 bp homology to the sequence directly downstream of the *parC* gene were used to amplify *mKate2* with *parC* homology tails from pEH04. These were as follows:

5′GTGTTGAGATCGACTCTCCTCGCCGTGCCAGCAGCGGTGATAGCGAAGAGTCTGGTTCTGGTTCTGGTTCTGGTTCTGGTTCTGGT GTGAGCGAGCTGATTAAGGAG 3′.

5′TCATCCGGCGTTCCTTGCAAGCGGGAGGAAACAGCGCCCTCCCCGGCATA TTACGCCAAGCTTGTGTAGGCT 3′.

Next, the PCR-product with flanking tails was electroporated into AB1157 cells, and homologous recombination facilitated by induction of plasmid pRed/ET (GeneBridges) as described in^34^.

The construct was verified by sequencing to be inserted at the correct position on the chromosome (at the endogenous *parC* gene) and to contain an amino acid linker sequence (Ser-Gly)^6^ between the C-terminal of ParC and the start of mKate2.

To obtain the strains used here with combinations of fluorescent constructs and/or mutations, P1 transduction^35^ and FLP recombinase (pCP20) was used^36^.

### Cell growth

Cells were grown at 28 °C in AB minimal medium^37^ supplemented with 0.4% sodium acetate, 1-μg ml^−1^ thiamine, 80-μg ml^−1^ threonine, 20-μg ml^−1^ leucine, 30-μg ml^−1^ proline, 22-μg ml^−1^ histidine and 22-μg ml^−1^ arginine (acetate medium). The doubling time (τ) was found by optical density (OD) measurements. Cells were grown to OD ∼ 0.15 (early exponential phase) at which time they were prepared for flow cytometry analysis or fluorescence microscopy. For experiments with Ciprofloxacin, EH34 cells were treated with 0.1 μg/ml for 45 min prior to imaging.

### Flow cytometry and cell cycle analysis

Exponentially growing cells were fixed in ethanol or treated with 300-μg/m rifampicin and 10-μg/ml cephalexin to inhibit replication initiation^38^ and cell division^39^, respectively. Growth of drug-treated samples continued for 3–4 generations, after which they were fixed in ethanol. Drug-treated cells ended up with an integral number of chromosomes^38^, which represents the number of origins at the time of drug treatment (replication run-out). Flow cytometry was performed as previously described^40^ using an LSR II flow cytometer (BD Biosciences) and FlowJo 7.2.5 software. Cell cycle parameters, numbers of origins and replication forks per cell were obtained by analysis of the DNA distributions obtained by flow cytometry as described^27^.

### Fluorescence microscopy imaging

For fluorescence microscopy exponentially growing cells were immobilized on an agarose pad (1% agarose in phosphate-buffered saline) and covered with a #1.5 coverslip. Images were acquired with a Leica DM6000 microscope equipped with a Leica EL6000 metal halide lamp and a Leica DFC350 FX monochrome CCD camera. Phase contrast imaging was performed with an HCX PLAPO 100×/1.40 NA objective. Narrow band-pass filter sets (CFP: Ex BP436/20, Em BP480/40, YFP: Ex BP510/20, Em BP560/40, Cy3: Ex BP545/30, Em BP610/75) were used for fluorescence imaging.

During image acquisition, saturated pixels were avoided. The raw images were saved for further image processing (see below).

### Image processing and analysis

Imaging adjustments (brightness and contrast) were performed in Image J or Fiji software. We used the public domain Coli-Inspector project to obtain fluorescence intensity profiles of the cells and to do vertical plotting of fluorescence and phase contrast images of cells. Coli-Inspector runs under ImageJ/Fiji in combination with the plugin ObjectJ (http://simon.bio.uva.nl/objectj/). The average fluorescence intensity profile of cells was plotted against the cell long axis, in groups of increasing cell length, as described^41^. Vertical plotting of cells was done in the order of gradual increase in cell length. Age classes of cells were defined by the cell length, assuming that length increases linearly.

We used a Python-based script developed in our group for measurements of distances between neighboring spots/foci that are registered in two different fluorescence channels. The script outputs all registered distances (in this case distances between SeqA, ParC and SSB) per cell, and these values were used to calculate average distances from at least three separate experiments. Image processing for automated analysis using this script was performed in Image J using the following tools: (i) Background subtraction with default Rolling disk (diameter 10 pixels), (ii) Deconvolution using the Richardson-Lucy algorithm (100 iterations), (iii) Median filter, ((iv) thresholding by Max Entropy (see^22^ for details). The positive correlation and p-value for increase in SeqA-ParC distances in Ciprofloxacin treated cells was calculated using a paired, one-tailed T-test on average distances from three independent experiments.

## Supplementary Information

Supplementary Information.
