# Supplementary material for: Topoisomerase IV tracks behind the replication fork and the SeqA complex during DNA replication in Escherichia coli
Source: Sci Rep. 2021 Jan 12;11:474. doi: 10.1038/s41598-020-80043-4 (PMC7803763; doi:10.1038/s41598-020-80043-4)
Supplement: Supplementary file 1 — Supplementary Information. [file 41598_2020_80043_MOESM1_ESM.pdf]

Supplementary material

Topoisomerase IV tracks behind the replication fork and the SeqA complex during DNA replication in *Escherichia coli*

Emily Helgesen[1,2]*, Frank Sætre[1,#] and Kirsten Skarstad[1]

[1] Department of Microbiology, Oslo University Hospital, Oslo, Norway

[2] Department of Clinical and Molecular Medicine, Faculty of Medicine and Health Sciences, Norwegian University of Science and Technology, Trondheim, Norway

[#] Current address: Department of Pathology, Institute of Clinical Medicine, University of Oslo, Oslo, Norway

Table S1: Table of strain names, relevant features and sources.

**Strain name**	**Relevant features**[a]	**Source**
AB1157	Wild type strain	(1)
GL224	MG1655 *SSB-CFP::cat* in *lamB*	A. Wright and G. Leung
MG1655 *seqA-YFP*	MG1655 *seqA-YFP::cat*	(2)
BS30	AB1157 *parC-mKate2::cat*	This work
BS36	AB1157 *seqA-YFP parC-mKate2::cat*	This work
EH29	AB1157 *seqA-YFP parC-mKate2 SSB-CFP::cat*	This work
LZ3099	C600 *gyrA*$^{S83L+D87Y}$	(3)
EH32	AB1157 *gyrA*$^{S83L+D87Y}$	This work
EH34	AB1157 *seqA-YFP parC-mKate2::cat gyrA*$^{S83L+D87Y}$	This work

[a] Description of strain constructions in Materials and Methods

Figure S1: Time-lapse imaging of cells containing SSB-CFP, ParC-mKate2 and SeqA-YFP.

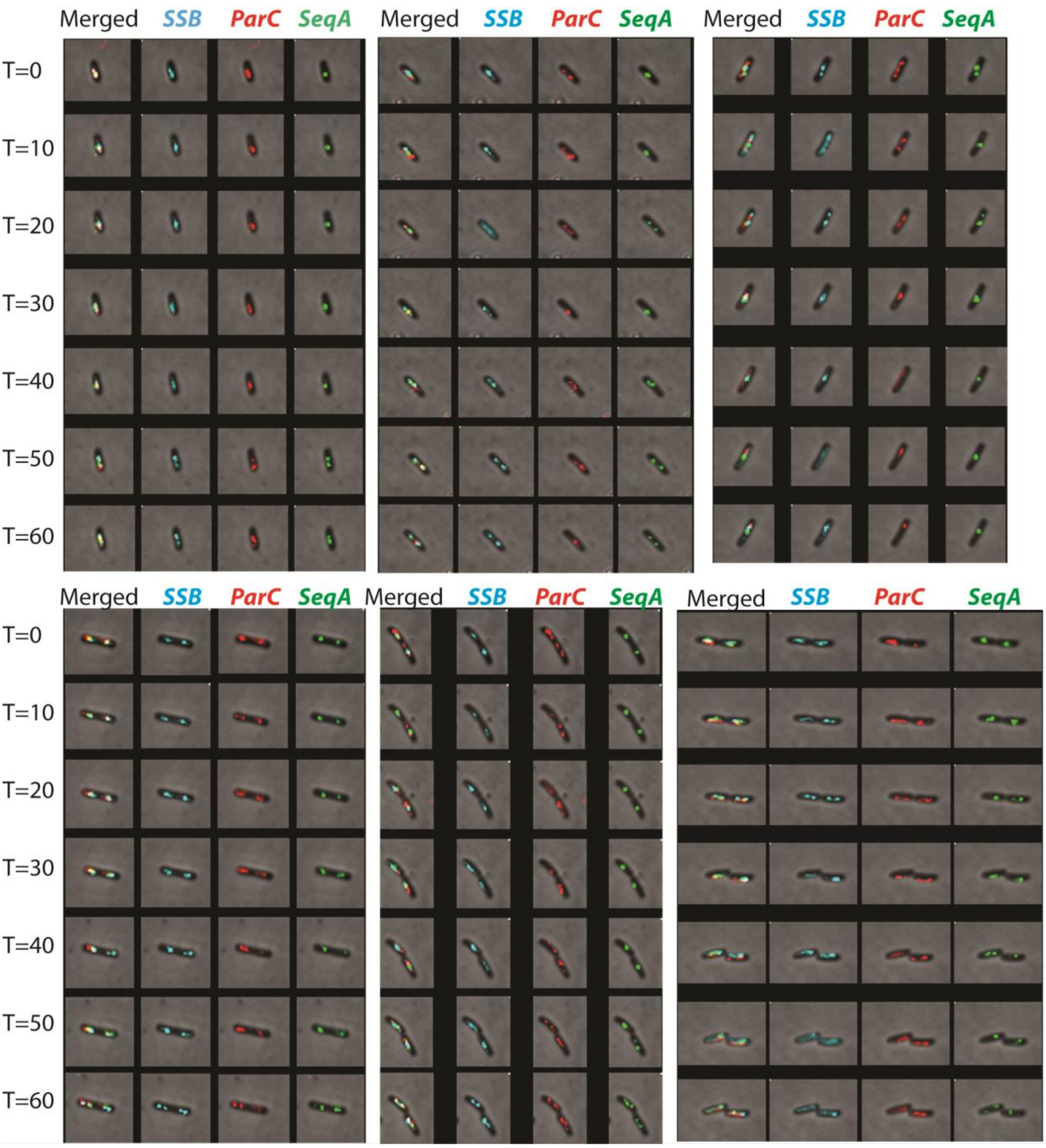

Fig S1: Images of representative cells from time-lapse imaging of strain EH29 (**SSB-CFP**, **ParC-mKate2** and **SeqA-YFP**). The cells were grown in acetate medium at 28 °C to OD~0.15 and dispensed onto a microscopy slide containing an agarose pad (1%) with acetate medium. The pad was covered with a glass coverslip, and the cells were imaged every 10 minutes for 1 hour.

Figure S2: Flow cytometry histograms of EH29 cells

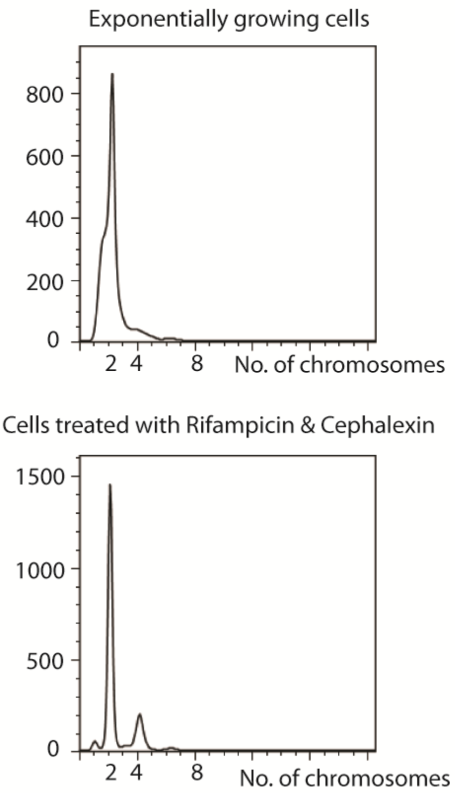

Fig S2: Flow cytometry histograms showing the DNA content of exponentially growing cells (top panel) and cells treated with Rifampicin and Cephalexin to produce a run-out of DNA replication (bottom panel) of the strain EH29. The cells were grown at 28°C in acetate medium (see Methods for medium composition).

1. Howard-Flanders, P., Simson, E. and Theriot, L. (1964) A locus that controls filament formation and sensitivity to radiation in *Escherichia coli* K-12. *Genetics*, **49**, 237-246.
2. Babic, A., Lindner, A.B., Vulic, M., Stewart, E.J. and Radman, M. (2008) Direct visualization of horizontal gene transfer. *Science (New York, N.Y.)*, **319**, 1533-1536.
3. Morgan-Linnell, S.K. and Zechiedrich, L. (2007) Contributions of the combined effects of topoisomerase mutations toward fluoroquinolone resistance in *Escherichia coli*. *Antimicrobial agents and chemotherapy*, **51**, 4205-4208.
